# An Algorithm to detect balancing of iterated line sigraph

**DOI:** 10.1186/s40064-015-1499-0

**Published:** 2015-11-17

**Authors:** Deepa Sinha, Anshu Sethi

**Affiliations:** 1 South Asian University Akbar Bhawan, Chanakyapuri, New Delhi 110 021 India; 2 Center For Mathematical Sciences, Banasthali University, Banasthali, 304 022 Rajasthan India

**Keywords:** Sigraph, Line sigraph, Balanced sigraph, Iterated line sigraph, Negative section, Network, Encryption and decryption

## Abstract

A *signed* *graph* (or *sigraph* in short) *S* is a graph *G* in which each edge *x* carries a value $$s(x) \in \{+1,
								-1\}$$ called its *sign* denoted specially as $$S = (G,
								s)$$. Given a sigraph *S*,  *H* = *L*(*S*)   called the *line sigraph* of *S* is that sigraph in which edges of *S* are represented as *vertices*, two of these vertices are defined to be adjacent whenever the corresponding edges in *S* have a vertex in common and any such edge *ef* is defined to be *negative* whenever both *e* and *f* are negative edges in *S*. Here *S* is called *root sigraph* of *H*. *Iterated signed line graphs* $$L^k(S)$$ = $$L(L^{k-1}(S)),$$ *k* $$\in$$ $$\mathbb
								{N}$$, *S*:= $$L^0(S)$$ is defined similarly. In this paper, we give an algorithm to obtain iterated line sigraph and detect for which value of ‘*k*’ it is *balanced* and determine its complexity. In the end we will propose a technique that will use adjacency matrix of *S* and adjacency matrix of $$L^k(S)$$ which is balanced for some ‘*k*’ as a parameter to encrypt a network and forward the data in the form of balanced $$L^k(S)$$ and will decrypt it by applying inverse matrix operations.

## Background

For standard terminology and notation in *graph theory* we refer to Harary (1969), West (1996) and Zaslavsky (1981, 1982) for *sigraphs* and Cormen et al. (2011) and Golumbic (2004) for *algorithms*. Throughout the text, we consider finite, undirected graph with no loops or multiple edges. By an (*n*, *e*) graph *G* we mean a graph having *n* *vertices* and *e* *edges*; *n* is called the *order* and *e* is called the *size* of *G*. In computer science domain, any graph *G* is observed as a network by computer scientists where vertices are taken to be *nodes* and edges to be taken as *links*.

A *signed* *graph* (or *sigraph* in short) Zaslavsky (1982) is an ordered pair $$S = (S^u, \sigma
							)$$, where $$S^u =
							(V,E)$$ is a graph called the *underlying graph* of *S* and $$\sigma : E
							\rightarrow \{+,-\}$$ is a function from the edge set *E* of $$S^u$$ into the set $$\{+,-\}$$, called the *signature* (or *sign* in short) of *S*.

**Fig. 1:**
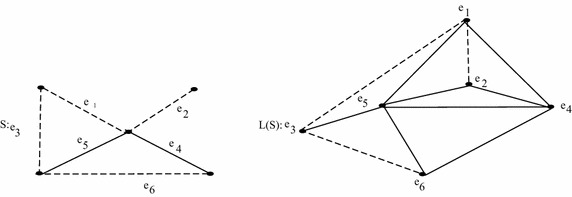
Example showing sigraph and its line sigraph

For a sigraph *S*,  Behzad and Chartrand (1969) define its *line sigraph*, *L*(*S*) as the sigraph in which the edges of *S* are represented as vertices, two of these vertices are defined adjacent whenever the corresponding edges in *S* have a vertex in common, any such edge *ef* is defined to be negative whenever both *e* and *f* are negative edges in *S*. A given sigraph *S* is a linesigraph if it is isomorphic to the linesigraph *L*(*T*) of a sigraph *T*. Here *T* is called the line root of *S*. A sigraph *S* and its line sigraph *L*(*S*) is shown in Fig. 1.

A sigraph *S* is *sign-compatible* Sinha (2005); Sinha and Sethi 2015) if there exists a marking $$\mu$$ of its vertices such that the end vertices of every negative edge receive ‘−’ signs in $$\mu$$ and no positive edge in *S* has both of its ends assigned ‘−’ sign in $$\mu
							.$$ In other words, a sigraph is sign-compatible if and only if its vertices can be partitioned into two subsets $$V_1$$ and $$V_2$$ such that the all-negative subsigraph of *S* is precisely the subsigraph induced by exactly one of the subsets $$V_1$$ and $$V_2.$$ Every line sigraph is sign-compatible. However, not every sign-compatible sigraph need be line sigraph.

An adjacency matrix for a network with *n* vertices and no parallel edges is an $$n \times
							n$$ symmetric matrix such that

$$\begin{aligned}
							a_{ij} = \left\{ \begin{array}{ll} 1 &{} \quad \text {if}\;(i,
							j)\; \text {is a solid line}\\ -1 &{} \quad \text {if}\; (i,
							j)\; \text {is a dotted line}\\ 0 &{} \quad \text {if} \;(i=j)
							\end{array} \right. \end{aligned}$$

A *cycle* in a sigraph *S* is said to be *positive* if the product of the signs of its edges is positive or, equivalently, if the number of negative edges in it is even. A cycle which is not positive is said to be *negative.* A sigraph is said to be *balanced* if every cycle in it is positive (Harary 1953; Cartwright and Harary 1956; Acharya and Acharya 1986). The following characterization of balanced sigraphs is well known:

### Theorem 1

(Harary and Kabell 1980)* A sigraph is balanced if and only if there exists a partition of its vertex set into two subsets, one of them possibly empty, such that every positive edge joins two vertices in the same subset and every negative edge joins two vertices from different subsets.*

For any positive integer *k*, the *k*th *iterated line sigraph* $L^k(S)$ of *S* is defined (see Gill and Patwardhan 1983) recursively as follows:

$$L^0(S)$$ = *S*, $$L^k(S)$$ = $$L(L^{k-1}(S)).$$

**Fig. 2:**
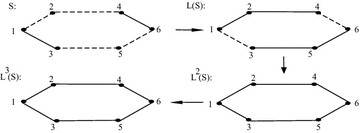
Example showing sigraph and its line sigraph upto 3 iterations

A sigraph *S* and its iterated line sigraph $L^k(S)$ is shown in Fig. 2.

By a *negative section* (Gill and Patwardhan 1981) of a subgraph $S'$ of a sigraph *S* we mean a maximal edge-induced subgraph in $S'$ consisting of only the negative edges of *S*; in particular, a negative section in a cycle of *S* is essentially a maximal all-negative path in the cycle or the whole cycle itself. Thus, a cycle is positive if and only if it has an even number of negative sections of odd length.

In this paper, we are going to introduce a new method for encoding and decoding of data using network as sigraphs and basic properties of matrices. For the purpose of network security, adjacency matrix of *S* will be considered as basis of information which is to encrypted to adjacency matrix of balanced $L^k(S)$ for some ‘k’ to assure confidentiality, integrity and authentication of transmitted data.

The following result gives a characterization of sigraphs whose line sigraph *L*(*S*) is balanced:

### Theorem 2

(Sinha 2005)* For any sigraph S*,  *L*(*S*) *is balanced if and only if the following conditions hold on S*: 

1. *For any cycle* *Z in* *S*; 
  - *If Z* *is all negative then* *Z* *is of even length;*
  - *If Z* *negative sections of non-zero even length inis heterogenous then there is an even number of* *Z*; 
2. *For any vertex* *v* *in* *S,  if the degree exceeds two then there is at most one negative edge incident at* *v*.

## Balanced iterated signed line graphs

In this section, we extend Theorem 2 to any iterated line sigraph $$L^k(S),$$ $$k \in \mathbb
							{N}.$$

### Theorem 3

(Sinha 2005; Sinha and Acharya 2015) *For any sigraph* *S,  and for any positive integer k,* $L^k(S)$ *is balanced if and only if the following conditions are satisfied by* *S* : 

1. *For any cycle* *Z* in *S*; 
  - *If* *Z is all negative then* *Z* i*s of even length;*
  - *If* *Z* *is heterogenous then the number of negative sections of odd(even) length greater than* *k* *is even if* *k* *is even(odd); and*
2. *For any vertex* *v* *in* *S,  if* $$d(v) >
											2$$ *then* $$d^-(v) <
											3,$$ *and if* $$d^-(v) = 2,$$ *then length of any negative section through* *v* *is at most* *k*.

**Fig. 3:**
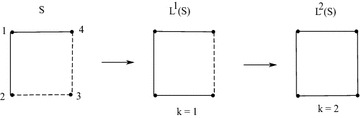
For a sigraph *S*, $L^1(S)$ is not balanced and $L^2(S)$ is balanced

A sigraph *S* and its iterated line sigraph such that $L^1(S)$ is not balanced and $L^2(S)$ is balanced is shown in Fig. 3.

### Numerical interpretation of above characterization

Following is the numerical analysis for the sigraphs shown in Fig. 3. The adjacency matrix corresponding to sigraph *S* is defined as follows:

$$\begin{aligned}
								A(S) = \left[ \begin{array}{cccccccccccc} 0 &{} 1
								&{} 0 &{} 1 \\ 1 &{} 0 &{} -1
								&{} 0 \\ 0 &{} -1 &{} 0 &{} -1\\ 1
								&{} 0 &{} -1 &{} 0 \end{array}\right]
								\end{aligned}$$

Following procedure is implemented to obtain an iterated line sigraph from a given sigraph and check whether this iterated line sigraph is balanced or not:

 Enter the number of nodes, i.e., *n*. Input $$n \times
								n$$ adjacency matrix with respect to given sigraph. The adjacency matrix takes the entries as 0, 1 and $$-$$1 for no edge, positive edge and negative edge respectively. Now we have to find the line sigraph of this sigraph Sinha and Sethi (2015). For this, first we populate EdgeList (list of all edges in the given sigraph) by assigning each edge an index. We then search for non-zero entries in adjacency matrix and then corresponding to each such entry, say (*i*, *j*)th entry, we assign the edge number as 1, 2,3,...

Then for each edge we check adjacent edges from the EdgeList prepared and see if they have the common vertex. The sign of the vertex depends on the sign of edge (*i*, *j*)th in *S*. If edge (*i*, *j*) is positive then corresponding vertex would be positive otherwise it would be negative. This way new matrix of *L*(*S*) is computed.

Since we have to compute $L^k(S),$ it means we have to repeat the above conversion of converting sigraph to its line sigraph ‘*k*’ times such that number of edges of first iterated line sigraph now becomes number of vertices for the second iterated line sigraph and output matrix of first iterated line sigraph is now the input matrix for the required second iterated line sigraph and so on.

Similarly, $L^k(S)$ is computed.

To check 1(a) condition, i.e. if cycle is negative, we will find all cycles in the given sigraph. For each cycle we calculate the length of the path and count all negative entries corresponding to this path. If path length is equal to half of the negative values, it means that cycle is homogenous i.e. it contains all the negative edges otherwise heterogenous. If cycle is homogeneous, then count must be even otherwise we terminate the procedure and say $L^k(S)$ is not balanced.

For the adjacency matrix of *S*, length of the path 1-2-3-4 of cycle is 4 and half count of all negative entries is = 4/2 i.e. 2. Since length of path of cycle does not match the number of negative entries, therefore, cycle is not homogenous and we check the next condition.

To check 1(b) condition we first find a cycle. Then we search for positive edge in the path. If we found any positive edge it means the cycle is heterogeneous. Now we count $-$1 entries in the path. If it contains only one negative edge proceeded with a positive edge consecutively in the path, it means it has negative section. Now we count $-$1 entries and check if it is greater than ‘*k*’ and of odd length, we update NbOddNegativeSections (number of negative sections of odd length) by 1 and if it is greater than ‘*k*’ and of even length then we increment NbEvenNegativeSections (number of negative sections of even length) by 1. This way total number of negative sections of odd length and even length greater than ‘*k*’ is calculated. Now we check if this count NbOddNegativeSections is even when ‘*k*’ is even and NbEvenNegativeSections is odd when ‘*k*’ is odd. If both the condition are satisfied we check the next condition otherwise we terminate the procedure.

For the adjacency matrix of *S*,  it has 2 negative sections, 1-2-3 of length 2 and 4-5-6 also of length 2. Take *k* = 1 i.e. ‘*k*’ is odd. Count number of negative sections of even length $>$ *k*, which is 2 but according to the theorem this count must be odd. Given condition does not hold true for *k* = 1, therefore for given sigraph *S*,  $L^1(S)$ is not balanced. Take *k* = 2 i.e. ‘*k*’ is even, count number of negative sections of odd length $>$ *k*, which is 0 in our case and is even. Thus, given condition holds true for *k* = 2 and we proceed to check the next condition.

To check 2 condition we calculate degree of each vertex by counting non-zero entry in each row and negative degree by counting $$-$$1 entries in each row. Then for each vertex we check if degree is $$>$$2 then negative degree must be $$\le$$ 3 and if $$d^-(v) =
								2,$$ then we calculate again the length of each negative section incident at *v* by applying the same procedure as calculated in condition 1(a).

For the adjacency matrix of *S*,  there exists no vertex where degree $>$2, therefore, given condition is also satisfied for given *S*. Thus, we can say that for given sigraph *S*, $L^1(S)$ is not balanced and $L^2(S)$ is balanced.

### Algorithm to detect balancing of iterated line sigraph

The algorithm to detect balancing of line sigraph is based on the characterization given by Acharya and Sinha (2003). To detect balancing of iterated line sigraph $L^k(S)$ of *S*, we have to first obtain $L^k(S)$ from *S*. Following is the algorithm to $L^k(S)$ of a given sigraph *S*:

#### Algorithm to convert a sigraph to its iterated line sigraph SigraphtoIteratedLinesigraph (vertex, n, k)

Here *vertex* is the input matrix and *n* is the number of vertices of a given sigraph. *k* denotes number of iterations whose *k*th iterated line sigraph $L^k(S)$ is to be computed.

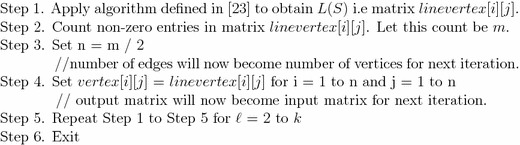



**Note**: Since, if we have *n* number of vertices, then for *k* = 1, there are maximum of $n(n-1) / 2$ edges, which in turn becomes number of vertices for the next iteration and so on. For *k* =2, the number of vertices will be of order $n^2$ and correspondingly number of edges will be of order $n^4$ and so on. Thus, *k*th iteration will have maximum $n^k$ number of vertices and $n^{2k}$ number of edges.

**Complexity of computation involved in above algorithm**

In Step 1, we have applied the algorithm defined in Sinha and Sethi (2015) to obtain *L*(*S*), Thus complexity of this step = $O(n^2).$

In Step 5, we have to compute $L^k(S)$ and we have to repeat Step1, $k-1$ times, therefore,

Complexity of this step = $$n^{k-1} \times
									O(n^2)$$ = $$O(n^{2(k-1)}),$$ where *k* denotes number of iterations.

Hence complexity of computation involved in above algorithm is $O(n^{2(k-1)}),$ where *n* is number of vertices in *S*.

**Numerical interpretation**

The adjacency matrix corresponding to sigraph $L^1(S)$ and $L^2(S)$ is defined as follows:

$$\begin{aligned} A\left( L^1(S)\right) =
									\begin{bmatrix} 0&1&1&0 \\
									1&0&0&1 \\
									1&0&0&-1 \\
									0&1&-1&0 \end{bmatrix} \quad
									\mathrm{and} \quad A\left( L^2(S)\right) = \begin{bmatrix}
									0&1&1&0 \\
									1&0&0&1 \\
									1&0&0&1 \\
									0&1&1&0 \end{bmatrix}
									\end{aligned}$$

#### Main algorithm

Following is the algorithm to detect balancing of an iterated line sigraph:

$$\begin{aligned}
									\mathbf{IteratedLineSigraphBalance \,\, ( vertices, n, k)}
									\end{aligned}$$

Here *vertices* is the input matrix and *n* is the number of vertices of a given sigraph. *k* denotes number of iterations. *NbPositiveEdges*, *NbNegativeEdges* and *NbTotalEdges* represents total number of positive edges, negative edges and total edges incident to each vertex.

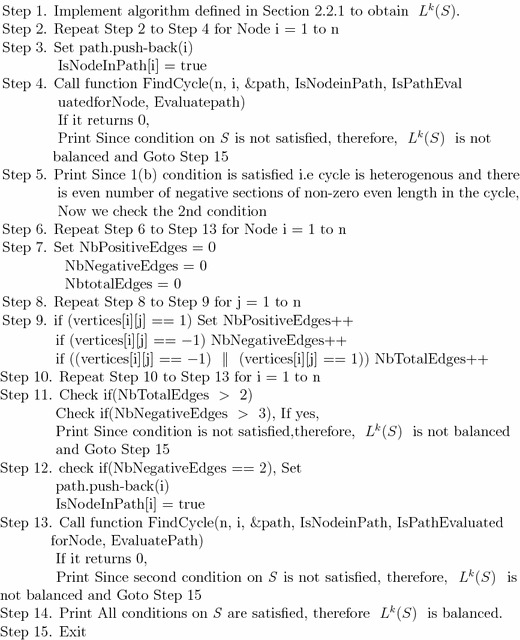



Here *FindCycle* is a function used to find all cycles within a given sigraph from its adjacency matrix. *StartNode* represents first node from where the cycle start. *PathUptilNow* is a vector used to represent the position of the node till the vertex is traversed. *IsNodeInPath* is a boolean varible used to detect whether nodes is already in the path or not. *IsPathEvaluatedForNode *is also a boolean variable used to find whether nodes for which path is already calculated. *Evaluate = 0* is a function to evaluate path. *cur-node* represents the current node which is traversed.

**Function FindCycle (n, StartNode, Pathuptilnow, IsNodeinPath, IsPathEvaluatedforNode, Evaluate = 0)**

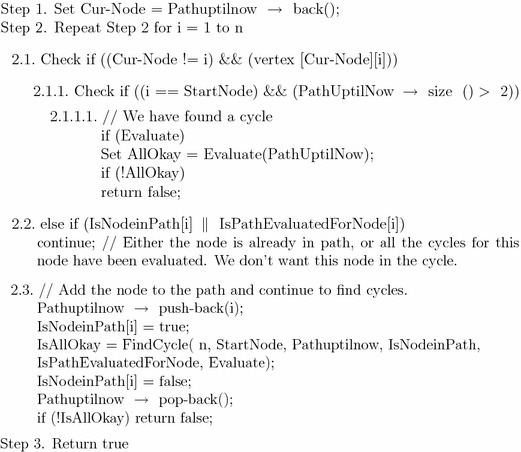



Above function outputs a path of the cycle. Here Function *EvaluatePath* is used to detect whether cycle is homogenous or heterogenous. If it is heterogenous, then it counts number of negative sections of odd(even) length greater than *k* is even if *k* is even(odd) or not. *NbEdgesInSection* denotes total number of edges in each section. *NbNegativeSections* represents total number of negative sections. *NbEvenNegativeSections* and *NbOddNegativeSections* denotes number of negative sections of even and odd length respectively.

**Function evaluate (path)**

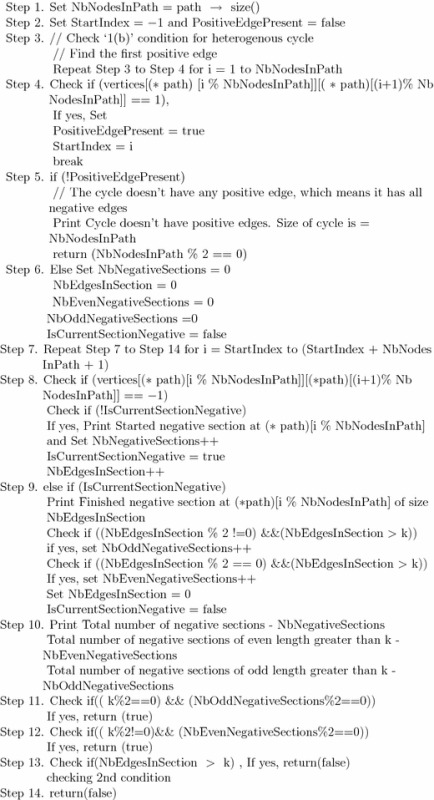



**Complexity of computation involved in above algorithm**

In Step 1, we compute $L^k(S)$ as defined in "Main algorithm", therefore,

Complexity of this step = $O(n^{2(k-1)}).$

In Step 2, each node of the graph is traversed and for each node we have to find cycle i.e. Step 4 is called ‘*n*’ times. For finding cycle, we have to call for function EvaluatePath() where each vertex is again traversed. Thus complexity of this step = $$O(n) \times n \times
									n$$ = $$O(n^3).$$

For the function EvaluatePath(), in Step 3 and Step 4 we first calculate first positive edge and for this we have to traverse each node of the path, Thus, Complexity of this step = *O*(*n*).

In Step 7, for finding number of negative sections and length of each section we repeat Step 7 to Step 12 and it is repeated for each cycle, therefore, Complexity of this step = $O(n) \times n$ = $O(n^2).$

For checking the 2 condition we calculate positive, negative and total edges incident to each vertex, for this, we follow Step 6 to Step 9 in the main algorithm and for this we traverse again $n \times n$ matrix, Thus, complexity of this step = $O(n^2).$

In Step 10 to Step 13, we check total degree and negative degree of each vertex, therefore, Complexity of this step = *O*(*n*).

In Step 13, we call the function FindCycle(), such that whole procedure is again repeated, therefore, Complexity of this step = $$O(n) \times n \times
									n$$ = $$O(n^3).$$

Total complexity = $$O(n^{2(k-1)})$$ + $$O(n^3)$$ + *O*(*n*) + $$O(n^2)$$ + $$O(n^2)$$ + *O*(*n*) + $$O(n^3)$$ = $$O(n^3 +
									n^{2(k-1)}).$$

**Note:** Our main aim is to only detect balancing of iterated line sigraph, therefore, complexity to obtain iterated line sigraphs can be neglected.

Thus Total complexity for detecting balancing of iterated line sigraph = $O(n^3)$ + *O*(*n*) + $O(n^2)$ + $O(n^2)$ + *O*(*n*) + $O(n^3)$ = $O(n^3).$

Hence complexity of computation involved in above algorithm is $O(n^3),$ where *n* is number of vertices in *S*.

**Correctness of the above algorithm**

An algorithm is said to be correct if for every input data that satisfies some conditions-called the precondition of the algorithm, the output data satisfy a certain predefined condition-called the post condition of the algorithm. A graph algorithm depends upon number of vertices and edges in a graph and inter/intra relationship between these two features. Here, we will discuss an approach based on the adjacency matrices of the given sigraph to prove or disprove the correctness of the above proposed algorithm.

##### Example 1

**Fig. 4:**
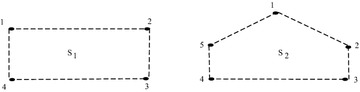
Example showing homogeneous cycle of odd and even length

Consider the sigraphs shown in Fig. 4.

The adjacency matrix corresponding to sigraph $S_1$ and $S_2$ is defined as follows:

$$\begin{aligned} A(S_1)= \begin{bmatrix}
										0&-1&0&-1 \\
										-1&0&-1&0 \\
										0&-1&0&-1 \\
										-1&0&-1&0 \\ \end{bmatrix} \quad
										\mathrm{and} \quad A(S_2)= \begin{bmatrix}
										0&-1&0&0&-1 \\
										-1&0&-1&0&0 \\
										0&-1&0&-1&0 \\
										0&0&-1&0&-1 \\
										-1&0&0&-1&0 \\ \end{bmatrix}
										\end{aligned}$$

In Step 4, for $S_1$, we call for the function FindCycle() to find all cycles starting with initial node i=1. $S_1$ contain cycle 1-2-3-4-1 with path length 4(Path size) and count of negative edges(NbNodesInPath) as 4. Both the counts are same, therefore, cycle is homogenous and we have to check if this count is odd or even. If it is odd, then process is terminated after Step 4 in $O(n^3))$ steps otherwise next condition is checked. Similarly, for $S_2$, there exists cycle 1-2-3-4–5-1 with path length 5(Path size) and count of negative edges(NbNodesInPath) as 5. Both the counts are same, therefore, cycle is homogenous and we have to check if this count is odd or even. Since this count is odd, then process is terminated after Step 4 in $O(n^3))$ steps.

**Note:** For any sigraph to detect its balancing, we have to find cycle and complexity of finding cycle is $O(n^3)$, therefore, whole process must be implemented in atleast $O(n^3)$ steps.

##### Example 2

**Fig. 5:**
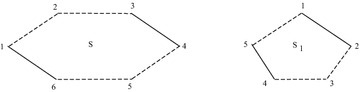
Illustration of another example

Consider another sigraphs as shown in Fig. 5.

The adjacency matrix corresponding to sigraph *S* and $S_1$ is defined as follows:

$$\begin{aligned} A(S)= \begin{bmatrix}
										0&-1&0&-1 \\
										-1&0&-1&0 \\
										0&-1&0&-1 \\
										-1&0&-1&0 \\ \end{bmatrix} \quad
										\mathrm{and} \quad A(S_1)= \begin{bmatrix}
										0&-1&0&0&-1 \\
										-1&0&-1&0&0 \\
										0&-1&0&-1&0 \\
										0&0&-1&0&-1 \\
										-1&0&0&-1&0 \\ \end{bmatrix}
										\end{aligned}$$

Consider a sigraph *S* as shown in Fig. 5. It has 2 negative sections, 1-2-3 of length 2 and 4-5-6 also of length 2. Take *k* = 1 i.e. *k* is odd. Now we have to count number of negative sections of even length $>$ *k*, which is 2 but according to the theorem this count must be odd. Since condition does not hold true for *k* = 1, therefore for given sigraph *S*, $L^1(S)$ is not balanced and process is terminated at Step 4 of main algorithm and implemented in $O(n^3)$ steps. If *k* = 2 i.e. *k* is even, then we have to count number of negative sections of odd length $>$ *k*, which is 0 in our case and is even. Therefore, condition holds true for *k* = 2 and we proceed to check the next condition. Since there does not exists any vertex with NbTotalEdges $>$2, therefore, given condition is also satisfied and process is again terminated in $O(n^3)$ steps.

Similarly, if we take sigraph $S_1,$ we have 2 negative sections, 5-1 of length 1 and 2-3-4 of length 2. Now it can be easily verified that for *k* =1 and *k* = 2, all conditions holds true.

## Conclusion and scope

In this paper, we have given an algorithmic approach to obtain iterated line sigraphs of a given sigraph and detect whether it is balanced or not in $O(n^3)$ steps. In this method, data is taken in the form of adjacency matrix with entries as 0, 1 and $-$1. Now since any matrix can be represented in the form of a network and vice-versa, we can apply encryption and decryption mechanism for a network through matrices. Algorithm is already defined to obtain a line sigraph from a given sigraph Sinha and Sethi (2015) and if we have algorithm to obtain line root sigraph of given sigraph, therefore, we can apply encryption and decryption algorithms with the additional condition that $L^k(S)$ is balanced for some ‘k’ to a network. There exists several techniques on encryption and decryption, but we have developed a new technique in which weak and strong relationships among nodes in a balanced network can be defined. For the purpose of security, we have used asymmetric key cryptography.

### System model

Asymmetric cryptography is used in this model. Two different keys, a secret key *d* and a public key *e* are defined. The public key *e* is used for encryption i.e. for converting adjacency matrix of *S* to adjacency matrix of $L^k(S)$ which is balanced for some ‘k’. Since we have a unique method for encryption, therefore, it can be published. Further, the secret private key *d* is used for decryption of adjacency matrix of $L^k(S)$ to adjacency matrix of *S*. Since we have many line root sigraphs of a given sigraph and we have to restrict our networks to obtain a unique line root sigraph, therefore, private key is used. Also, if labelling of vertices can be done by Lehot (1974), then only we obtain unique line root sigraph. The following model is used as an application to above algorithms.

**Fig. 6:**
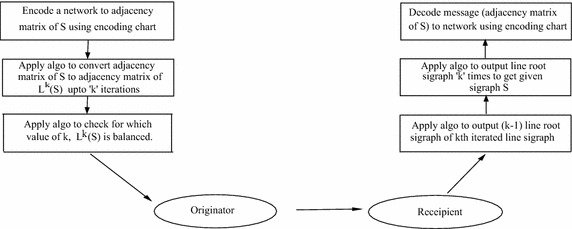
Application in Cryptography

The model used as an application to above algorithms can be referred in Fig. 6.

### Algorithms for system model

Apart from algorithms defined in "Algorithm to convert a sigraph to its iterated line sigraph SigraphtoIteratedLinesigraph (vertex, n, k)" and "Main algorithm", following are the algorithms that will be used in encryption and decryption mechanism.

#### Algorithm to detect a line sigraph and output its root sigraph

The algorithm to detect a line sigraph and output its line root sigraph is an extension of a paper named “An Optimal Algorithm to Detect a Line Graph and Output its Root Graph” by Lehot (1974).

To check whether a given sigraph is a line root sigraph or not we have to check two conditions:

1. The underlying graph is a line graph.
2. Given sigraph is sign-compatible.

If both the conditions are satisfied we say that line root sigraph exists and will print the new line root sigraph of *S*.

**Fig. 7:**
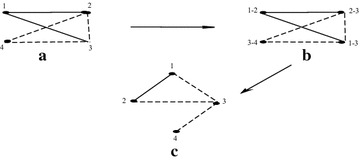
Step wise procedure to find line root sigraph

Figure 7a shows the sigraph corresponding to the input matrix. Now we have to find the line root sigraph of this sigraph. The step wise procedure in shown in Fig. 7. NewNode represents the node of the intermediate graph, i.e., Fig. 7b and Lookup represents if current index is mapped to which node. It shows the mapping between Fig. 7a, b. Since each node represents 2 points, maximum amount of numbers required is 2* max where max denotes maximum value of *n*.

NewGraph denotes the adjacency matrix of required line root sigraph and corresponding to this matrix Fig. 7c is plot.

Find the first non-zero entry in adjacency matrix i.e. *graph*[*i*] [*j*]. For the first edge (*i*, *j*) create a new node (*i*, 1, 2) $\rightarrow$ Node *i* named as pair (1, 2) i.e. in Fig. 7b.

Now start traversing the graph from this node. Also populate the final graph with this edge i.e. (1, 2). Now traverse through the graph and name other nodes. For each untraveled neighbor create a modified node for Fig. 7b. Find an *i*1 for new node. We want to find *j* as the first index *i*1,  we look if *j* is assigned to any node, do we have any edge. For e.g. if $j = 2$ and current nodes are (*a*, (1, 2)), (*b*, (2, 3)) and (*c*, (1, 4)) we check if current node has edge from *a* to *b* Ȯnce we have find *j* we want to find *k* as second index. If *j* and *k* are found we have found the naming of *i*1 and *i*2. Add *i*1 and *i*2 to NewNode. Make CurIdx minimum of (*CurIdx*, *j*, *k*). Record that *j* and *k* have been assigned to current node (*i*).

If pairing of edges can be done as given by Lehot (1974), then we say that graph is a line graph otherwise not a line graph and hence not a line root sigraph.

This pair would represent an edge in NewGraph. If this node has negative edge anywhere, set this edge as negative else positive. Also add this node to the queue as we want to travel its neighbor.

Next step we will check whether a given sigraph is sign-compatible or not. If sigraph is sign-compatible, line root sigraph exists and output the new modified graph otherwise line root sigraph does not exists.

##### Theorem 4

*Sigraph* *H is the line root of a sigraph* *S* *and unique line root of a sigraph* *S if and only*

1. $S^u$ *is a line graph and*
2. *Vertices of* *S can be assigned marks ‘+’ or *‘$-$ *’ such that both the ends of every negative edge receive ‘* $-$ *’ mark and the same is not true for any positive edge (i.e. S is sign-compatible) such that the end vertices of the positive path receiving ‘* $-$ *’ mark is of length exactly two.*

##### Theorem 5

*Line root sigraph* *H* *of a line sigraph* *S* *is unique if and only if*

1. *H* *is homogenous and all-negative or*
2. *Positive section is of length one and every negative edge has at least one negative degree.*

The final NewGraph is then plotted. This is the required line root sigraph. Since for a given line root sigraph we have many sigraphs, we have restricted ourselves to sigraphs based on Theorems 4 and 5.

It uses following functions:

Max denotes maximum number of vertices

Structure of Node : idx; // Primary and i1, i2; // The pair represents mapping between Fig. 7a, b. Vertex 1 in Fig. 7a is mapped to pair 1–2 in Fig. 7b. idx represents initial vertex and i1 and i2 represents corresponding mapping between the vertices.

IsTraversed is a function which represents whether node is travelled or not.

Lookup defines if the current index is mapped to which node. Now, since each node is given 2 numbers, the maximum amount of numbers required is 2*MAX.

NewGraph represents required line root sigraph.

**Figure:**
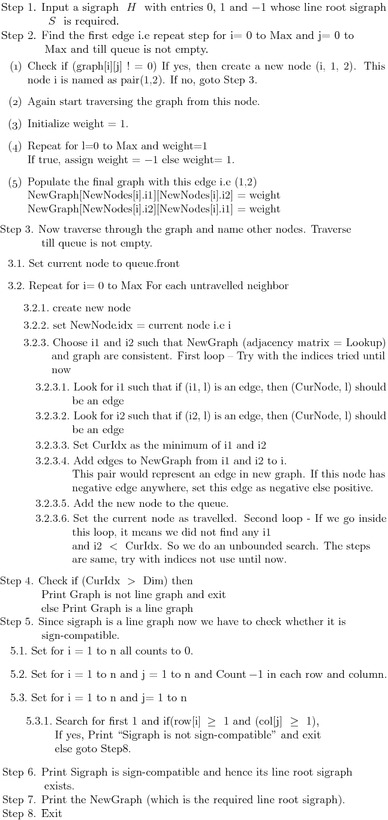


**Complexity of computation involved in above algorithm**

In Step 2, we have to find first non-zero entry (i.e. first edge) in adjacency matrix of order $n \times n$ and then corresponding to each such entry, say (*i*, *j*)th entry, we again have to traverse the graph to find its adjacent node and push the node in the queue.

Thus complexity of this step $=$ $O(n^3).$

In Step 3, since we have to traverse the graph and name all other nodes till queue is not empty. Queue contains the edges that are adjacent to first edge. This maximum number of edges is *e* = $n (n - 1)/2.$ Thus complexity of this step $=$ $O(e/n^3).$

In Step 5 we detect whether the given sigraph is sign-compatible or not. We count number of adjacent negative edges in adjacency matrix of order $n \times n$. Maximum number of edges can be $O(n^2).$ Thus complexity of this step $=$ $O(n^2).$

Total complexity $$=$$ $$O(n^3)$$ + $$O(e / n^3)$$ + $$O(n^2)$$ $$=$$ $$O(n^3).$$

Hence complexity of computation involved in above algorithm is $O(n^3),$ where *n* is number of vertices in *S* and *e* is the number of edges.

### Encoding chart

For a network with *n* number of vertices, we have $$n \times
								n$$ adjacency matrix encoded as shown in Fig. 8.

**Fig. 8:**
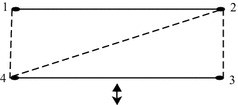
Encoding of a network to adjacency matrix

$$\begin{aligned}
								\begin{bmatrix} 0&1&0&-1 \\
								1&0&-1&-1 \\
								0&-1&0&1 \\
								-1&-1&1&0 \end{bmatrix}
								\end{aligned}$$

### Encryption algorithm

- Input the network which is to be encrypted. Then encode this network into adjacency matrix of *S*.
- Apply algorithm defined in Sinha and Sethi (2015) to convert a given adjacency matrix of *S* to adjacency matrix of *L*(*S*).
- Apply algorithm defined in Sect. 2.2.2 to detect if adjacency matrix of *L*(*S*) so produced is balanced for *k* =1. If it is balanced, then the adjacency matrix of *L*(*S*) is the encrypted data.
- Repeat algorithm defined in Sect. 2.2.1 to compute *k*th iterated line sigraph and algorithm defined in Sect. 2.2.2 to detect its balancing at each iteration. Stop the procedure where we get iterated line sigraph which is balanced for some *k*.
- The adjacency matrix for which $L^k(S)$ is balanced for some ‘k’ is the encrypted data.
- Now send the resultant adjacency matrix to the receiver in a linear format (i.e.  either column wise or row wise) with space between elements. *n* *n* $<$ Resultant matrix data $>$ *m* *m* where, *n* = number of vertices or nodes in the network *m* = number of edges in the network.

### Decryption algorithm

- Read the encrypted data and form the required matrices of order $m \times m.$
- Here the encrypted matrix is adjacency matrix of $L^k(S)$ which is balanced for some *k*.
- Apply “Algorithm to detect a line sigraph and output its root sigraph” defined in "Algorithm to detect a line sigraph and output its root sigraph" to obtain $L^{k-1}(S)$.
- Repeat the above step $(k-1)$ times to obtain back the resultant adjacency matrix of *S*.
- Decode adjacency matrix of *S* using encoding chart to generate the original network.

**Note:** Since we have restricted adjacency matrix of *S* to satisfy the property of $L^k(S)$ balancing for some ‘k’, therefore, our networks are restricted to have data with values 0, 1 and $-$1 and satisfying the balancing property of $L^k(S)$. The reading and writing of data can be done manually or by using file operation of any programming language( eg. C, C++...).

### Example

**Fig. 9:**
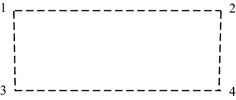
Network

Consider a network (Fig. 9):

Let

$$\begin{aligned}
								A(S) = \begin{bmatrix} 0&-1&-1&0 \\
								-1&0&0&-1 \\
								-1&0&0&-1 \\
								0&-1&-1&0 \end{bmatrix}
								\end{aligned}$$

be the adjacency matrix for the above network.

Compute adjacency matrix of *L*(*S*) corresponding to adjacency matrix of *S* by applying algorithm defined in Sinha and Sethi (2015). Thus the resultant matrix is adjacency matrix of *L*(*S*):

$$\begin{aligned}
								AL(S) = \begin{bmatrix} 0&-1&-1&0 \\
								-1&0&0&-1 \\
								-1&0&0&-1 \\
								0&-1&-1&0 \end{bmatrix}
								\end{aligned}$$

**Fig. 10:**
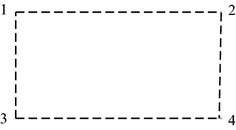
Network corresponding to *L* ^1^(*S*)

The corresponding graph is (Fig. 10):

Now we check whether this calculated $L^1(S)$ at *k* =1 is balanced or not. For this we apply algorithm defined in "Main Algorithm" from where it can be easily verified that it is balanced.

If we decide to transmit the data row wise, the data to be sent is ( the data are separated by space)

4 4 0 $$-$$1 $$-$$1 0 $$-$$1 0 0 $$-$$1 $$-$$1 0 0 $$-$$1 0 $$-$$1 $$-$$1 0 4 4

Suppose the received data is

4 4 0 $$-$$1 $$-$$1 0 $$-$$1 0 0 $$-$$1 $$-$$1 0 0 $$-$$1 0 $$-$$1 $$-$$1 0 4 4

From the data we have received we get the following matrix:

$$\begin{aligned}
								T' = \begin{bmatrix} 0&-1&-1&0 \\
								-1&0&0&-1 \\
								-1&0&0&-1 \\
								0&-1&-1&0 \end{bmatrix}
								\end{aligned}$$

isomorphic to some sigraph.

Now applying the decryption algorithm for $L^k(S)$ to *S* as shown in example we get the matrix as

$$\begin{aligned}
								T = \begin{bmatrix} 0&-1&-1&0 \\
								-1&0&0&-1 \\
								-1&0&0&-1 \\
								0&-1&-1&0 \end{bmatrix}
								\end{aligned}$$

which is equal to the adjacency matrix of *S*.
